# Urotensin-II-Targeted Liposomes as a New Drug Delivery System towards Prostate and Colon Cancer Cells

**DOI:** 10.1155/2019/9293560

**Published:** 2019-12-17

**Authors:** Silvia Zappavigna, Marianna Abate, Alessia Maria Cossu, Sara Lusa, Virginia Campani, Lorena Scotti, Amalia Luce, Ali Munaim Yousif, Francesco Merlino, Paolo Grieco, Giuseppe De Rosa, Michele Caraglia

**Affiliations:** ^1^Department of Precision Medicine, University of Campania “L. Vanvitelli”, Via L. de Crecchio, 7, 80138 Naples, Italy; ^2^Biogem Scarl, Institute of Genetic Research, Laboratory of Molecular and Precision Oncology, 83031 Ariano Irpino, Italy; ^3^Department of Pharmacy, University of Naples “Federico II”, Via D. Montesano, 49, 80131 Naples, Italy

## Abstract

Urotensin-II (UT-II) and its receptor (UTR) are involved in the occurrence of different epithelial cancers. In particular, UTR was found overexpressed on colon, bladder, and prostate cancer cells. The conjugation of ligands, able to specifically bind receptors that are overexpressed on cancer cells, to liposome surface represents an efficient active targeting strategy to enhance selectivity and efficiency of drug delivery systems. The aim of this study was to develop liposomes conjugated with UT-II (LipoUT) for efficient targeting of cancer cells that overexpress UTR. The liposomes had a mean diameter between 150 nm and 160 nm with a narrow size distribution (PI ≤ 0.1) and a doxo encapsulation efficiency of 96%. Moreover, the conjugation of UT-II to liposomes weakly reduced the zeta potential. We evaluated UTR expression on prostate (DU145, PC3, and LNCaP) and colon (WIDR and LoVo) cancer cells by FACS and western blotting analysis. UTR protein was expressed in all the tested cell lines; the level of expression was higher in WIDR, PC3, and LNCaP cells compared with LoVo and DU145. MTT cell viability assay showed that LipoUT-doxo was more active than Lipo-doxo on the growth inhibition of cells that overexpressed UTR (PC3, LNCaP, and WIDR) while in LoVo and DU145 cell lines, the activity was similar to or lower than that one of Lipo-doxo, respectively. Moreover, we found that cell uptake of Bodipy-labeled liposomes in PC3 and DU145 was higher for LipoUT than the not-armed counterparts but at higher extent in UTR overexpressing PC3 cells (about 2-fold higher), as evaluated by both confocal and FACS. In conclusion, the encapsulation of doxo in UT-II-targeted liposomes potentiated its delivery in UTR-overexpressing cells and could represent a new tool for the targeting of prostate and colon cancer.

## 1. Introduction

Urotensin-II (UT-II) has been described as the most potent vasoconstrictor, superior to other vasoactive molecules, such as endothelin-1, noradrenalin, and serotonin [1, 2]. In addition to vascular effects, UT-II and its receptor UTR have been found to be involved in the development of several diseases, such as cardiorenal diseases, heart failure, carotid atherosclerosis, and portal hypertension cirrhosis. Several studies have investigated the involvement of UT-II and UTR in human cancers [3]. In particular, UTR was overexpressed in colon [4, 5], bladder [6], and prostate [7] cancer cells. Recently, UTR has been proposed as a prognostic marker in prostate carcinoma since UTR expression was correlated with well-known pathological indicators of aggressive cancers, such as Gleason score. Interestingly, UTR was always expressed at low intensity in hyperplastic tissues and at high intensity in well-differentiated carcinomas (Gleason 2-3). Similarly, UT-II/UTR axis plays a key role in colon carcinogenesis [8]. UTR expression was low in normal colon tissues and increased in adenomas and colon cancers; moreover, our previous data suggest that UTR regulated motility and invasion of colon and bladder cancer cells [4, 6]. All these works clearly demonstrated that UTR represents a potentially useful target for innovative therapy against colon and prostate cancer, together with the revival of its previously reported modulators [9–11]. Liposomes are one of the most common vehicles proposed for drug targeting. In particular, liposomes with stealth properties allow the encapsulated drug to be addressed, such as doxorubicin (doxo), towards tissues characterized by vessels with an enhanced permeability of the endothelium, such as tumors. However, PEGylated liposomes containing doxo (marketed as Doxil® or Caelyx®) have demonstrated reduced toxicity although a higher delivery into the tumor has not been reported [12, 13]. To improve their target ability, liposomes have been functionalized with specific ligands or molecules, such as antibodies, peptides, and oligonucleotides, able to enhance the selectivity of liposomes towards cancer cells [14–16]. Several strategies can be approached for coupling ligand to the surface of stealth liposomes in order to achieve a high interaction of ligand with the receptor expressed on tumor cells. Among all commonly used approaches, PEG derivates include maleimide groups that react with cysteine residues or thiol groups on ligands to form stable thioether bonds between liposomes and ligands [17]. Thus, receptor ligands such as transferrin [18, 19], folic acid [13, 20, 21], or different monoclonal antibodies raised against tumor-associated antigens (TAA), including receptors [22, 23], have been largely investigated to target different cancer cells. Here, stealth liposomes were proposed as systems to target cancer cells overexpressing UTR and to promote drug delivery of an anticancer agent, i.e., doxorubicin, into colon and prostate cancer cells.

## 2. Materials and Methods

### 2.1. Materials

1,2-Dipalmitoyl-sn-glycero-3-phosphocholine (DPPC) and [N-(carbonyl-methoxypolyethylenglycol-2000)-1,2-distearoyl-snglycero-3-phosphoethanolamine, sodium salt] (DSPE-PEG_2000_) were purchased from Lipoid GmbH (Cam, Switzerland). 23-(Dipyrrometheneboron difluoride)-24-norcholesterol (Bodipy-cholesterol) and 1,2-distearoyl-sn-glycero-3-phosphoethanolamine-N-[maleimide-(polyethylene glycol)-2000] (ammonium salt) (DSPE-PEG_2000_-maleimide) were obtained from Avanti Polar Lipids (Alabaster, Alabama, USA). Doxorubicin hydrochloride (doxo), cholesterol (chol), sodium chloride, [4-(2-hydroxyethyl)piperazine-1-ethanesulfonic acid, N-(2-hydroxyethyl)piperazine-N′-(2-ethanesulfonic acid)] (Hepes), ethylenediaminetetraacetic acid (EDTA), iron thiocyanate (FeSCN_3_), sodium citrate, potassium chloride, citric acid, and Sepharose CL-4B were purchased from Sigma Chemical Co. (St. Louis, Mo, USA). All Fmoc-amino acids were purchased from GL Biochem (Shanghai, China) with their corresponding side chain protections for the solid-phase peptide synthesis (SPPS). Acetonitrile, methanol (HPLC degree), and chloroform (ACS grade) were obtained from Carlo Erba (Milan, Italy). RPMI, DMEM, and FBS were purchased from FlowLaboratories (Milan, Italy). Tissue culture plasticware was acquired from Becton Dickinson (Lincoln Park, NJ, USA). Rabbit antisera raised against *α*-tubulin and UTR were purchased from Santa Cruz Biotechnology (Santa Cruz, CA, USA). UT-II peptide was synthetized and purified by the research group of Prof. Paolo Grieco (Department of Pharmacy, University of Naples Federico II).

### 2.2. Peptide Synthesis

Peptide Cys-UT-II (H-Cys-Asp-c[Cys-Phe-Trp-Lys-Tyr-Cys]-Val-OH) was synthesized by adopting the solid-phase peptide synthesis (SPPS) using the Fmoc/*t*Bu orthogonal strategy, as reported elsewhere [24]. Upon cyclization procedure performed in solid phase [25], an additional Cys residue was added in the N-terminal by following standard procedures [26]. The product was purified and characterized by reverse-phase HPLC (RP-HPLC).

### 2.3. Liposome Preparation

Liposomes composed of DPPC/chol/DSPE-PEG_2000_ or DPPC/chol/DSPE-PEG_2000_-maleimide at a 65 : 30 : 5 molar ratio were prepared by hydration of a thin lipid film followed by extrusion. Briefly, lipids were dissolved in 1 mL of a mixture chloroform/methanol (2 : 1 v/v); the resulting solution was added to a 50 mL round-bottom flask, and the solvent was removed under reduced pressure by using a rotary evaporator (Laborota 4010 digital, Heidolph, Schwabach, Germany). Then, the resulting lipid film was hydrated with 1 mL of citrate buffer at pH 4 (150 mM sodium citrate; 150 mM citric acid), and the resulting suspension was gently mixed in the presence of glass beads until the lipid layer was removed from the glass wall; after that, the flask was left at 50°C for 2 h. The liposome suspension was then extruded at 50°C using a thermobarrel extruder system (Northern Lipids Inc., Vancouver, BC, Canada), passing repeatedly the suspension under nitrogen through polycarbonate membranes (5 times for each membrane) with decreasing pore sizes from 400 to 100 nm (Nucleopore Track Membrane 25 mm, Whatman, Brentford, UK). The external buffer was removed by ultracentrifugation (Optima Max E, Beckman Coulter, USA; rotor TLA 120.2) at 80,000 rpm, at 4°C for 40 min, and the liposomes were resuspended with 1 mL of phosphate buffer at pH 7.4 (140 mM sodium chloride; 25 mM of HEPES; 0.1 mM of EDTA). After preparation, liposomes were stored at 4°C. Each formulation was prepared in triplicate. For intracellular penetration studies, fluorescent-labeled liposomes containing 0.1% (w/w) of Bodipy-cholesterol were prepared similarly by adding the fluorescent cholesterol in the lipid mixture of DPPC/chol/DSPE-PEG_2000_ or DPPC/chol/DSPE-PEG_2000_-maleimide during the liposome preparation.

### 2.4. Preparation of Liposomes Conjugated with UT-II

Cys-UT-II (H-Cys-Asp-c[Cys-Phe-Trp-Lys-Tyr-Cys]-Val-OH) was conjugated with liposomes prepared with maleimide-modified polyethylene glycol. Briefly, the UT-II peptide (0.250 mM) was conjugated to liposomes containing DSPE-PEG_2000_-maleimide with gentle magnetic agitation at room temperature, overnight. The resulting liposomes, so-called LipoUT, were then chromatographed on a Sepharose CL-4B column in phosphate buffer at pH 7.4. All liposome preparations were stored at 4°C. The formulations were prepared in triplicate.

### 2.5. Preparation of Liposomes Encapsulating Doxorubicin

Doxo was encapsulated into liposomes *by remote loading* [27]. Briefly, the liposome suspension was combined with the doxo in a drug/lipid ratio of 0.2 (w/w) and then incubated at 50°C for 1 h. The un-encapsulated doxo was removed by ultracentrifugation at 80,000 rpm at 4°C for 40 min. After purification, the formulation was conjugated with UT-II, as described above, to obtain liposomes encapsulating doxo and conjugated with UT-II (LipoUT-doxo).

### 2.6. Determination of Lipid Concentration

The concentration of lipids present in the suspensions after preparation was determined using the Stewart assay [28]. Briefly, an aliquot of the suspension was added to a two-phase system consisting of an aqueous ammonium iron thiocyanate (FeSCN_3_) solution or iron thiocyanate solution (0.1 N) and CHCl_3_. The concentration of DPPC was obtained by measuring the absorbance at 485 nm into the organic layer with an ultraviolet-visible spectrophotometer (UV VIS 1204; Shimadzu Corporation, Kyoto, Japan). Quantification of DPPC was carried out by means of a calibration curve with standard DPPC samples.

### 2.7. Liposome Size and Zeta Potential

Dimensional analysis was performed at 20°C by photon correlation spectroscopy (PCS) (N5, Beckman Coulter, Miami, USA). Each sample was diluted in deionizer/filtered water (0.22 *μ*m pore size, polycarbonate filters, MF-Millipore, Microglass Heim, Italy) and analyzed with a detector at 90° angle. For measuring the particle size distribution, polydispersity index (PI) was used. For each formulation, the mean diameter and PI were calculated as the mean of three different batches. The average diameter and the size distribution of each formulation were determined. The results were expressed as liposome mean diameter (nm) and polydispersity index (PI). The zeta potential (*ζ*) of liposomes was performed by the Zetasizer Nano Z (Malvern, UK). Briefly, an aliquot of each sample (10 *μ*L) was diluted in filtered water and analyzed. The results were calculated by the average of 20 measurements.

### 2.8. Determination of Doxo Concentration

The amount of un-encapsulated doxo was determined as follows: 1 mL of liposome dispersion was ultracentrifugated at 80,000 rpm at 4°C for 40 min. Supernatants were carefully removed and doxo concentration was determined by using a UV/visible spectrophotometer at 480 nm. The results were expressed as encapsulation efficiency and were calculated as follows:

(1) $$\begin{array}{c} 1-\left[\frac{\left({\text{TS}}_{\text{doxo}}-{\text{AS}}_{\text{doxo}}\right)}{{\text{TS}}_{\text{doxo}}}\times 100\right], \end{array}$$

where TS_doxo_ is the theoretical doxo in the supernatant and AS_doxo_ is the actual doxo concentration in the supernatant.

### 2.9. UT-II Binding to the Liposome

The amount of UT-II linked to the liposomes was carried out by a high-performance liquid chromatography (HPLC) method. The HPLC system consisted of an isocratic pump (LC-10A VP, Shimadzu, Kyoto, Japan) equipped with a 7725i injection valve (Rheodyne, Cotati, USA) and SPV-10A UV-Vis detector (Shimadzu) set at the wavelength of 254 nm. The system was controlled by a SCL-10A VP System Controller (Shimadzu) connected to a computer. Chromatograms were acquired and analyzed by a Class VP Client/Server 7.2.1 program (Shimadzu, Scientific Instruments, Columbia, MD, USA). The quantitative analysis of UT-II was performed by reverse-phase chromatography (RP-HPLC) on a Luna 5 *μ*m C18 column (250 × 4.60 mm, 110 Å, Phenomenex, Klwid, USA) equipped with a security guard. The mobile phase was a mixture 40 : 60 (v/v) of acetonitrile and filtered water. The analyses were performed in isocratic condition, at a flow rate of the mobile phase of 1 mL/min and at room temperature. The amount of UT-II, present on the surface of nanocarriers, was determined as follows. Briefly, 1 mL of conjugated liposomes dispersion was ultracentrifugated (Optima Max E, Beckman Coulter, USA) at 80,000 rpm, at 4°C for 40 min. Supernatants were carefully removed, and the amount of UT-II unlinked to liposomes was determined by RP-HPLC. The amount of UT-II was calculated by means of a calibration curve with UT-II samples. The results have been expressed as follows:

(2) $$\begin{array}{c} 1-\left[\frac{\left({\text{UT-II}}_{\text{actual}}-{\text{UT-II}}_{\text{theor}}\right)}{{\text{UT-II}}_{\text{theor}}}\times 100\right], \end{array}$$

where UT-II_actual_ is the UT-II found in the supernatant and UT-II_theor_ is the theoretical UT-II added to the liposomes.

### 2.10. Cell Culture

Prostate (DU145, PC3, and LNCaP) and colon (WIDR and LoVo) cancer cell lines were obtained from American Type Culture Collection (ATCC; Rockville, MD, http://www.atcc.org). PC3, DU145, and WIDR cell lines were grown in DMEM, while LNCaP and LoVo were grown in RPMI 1640. Both cell media were supplemented with 10% heat-inactivated fetal bovine serum, 20 mM HEPES, 100 U/mL penicillin, 100 mg/mL streptomycin, 1% L-glutamine, and 1% sodium pyruvate. All the cells were cultured at a constant temperature of 37°C in a humidified atmosphere of 5% carbon dioxide (CO_2_).

### 2.11. Cell Proliferation Assay

After trypsinization, all the cell lines were plated in 100 *μ*L of medium in 96-well plates at a density of 2 × 10^3^ cells/well. One day later, the cells were treated with free doxo, plain Lipo, LipoUT, Lipo-doxo, and LipoUT-doxo at concentrations ranging from 20 *μ*M to 0.156 *μ*M.

Cell proliferation was evaluated by MTT assay. Briefly, cells were seeded in serum-containing media in 96-well plates at the density of 2 × 10^3^ cells/well. After 24 h incubation at 37°C, the medium was removed and replaced with fresh medium containing all developed formulations at different concentrations. Cells were incubated under these conditions for 72 h. Then, cell viability was assessed by MTT assay. The MTT solution (5 mg/mL in phosphate-buffered saline) was added (20 *μ*L/well), and the plates were incubated for further 4 h at 37°C. The MTT-formazan crystals were dissolved in 1N isopropanol/hydrochloric acid 10% solution. The absorbance values of the solution in each well were measured at 570 nm using a BioRad 550 microplate reader (BioRad Laboratories, Milan, Italy). Percentage of cell viability was calculated as

(3) $$\begin{array}{c} 100\%\;*\text{ (3)}\frac{\left(\text{absorbance of the treated wells}-\text{absorbance of the blank control wells}\right)}{\left(\text{absorbance of the negative control wells}-\text{absorbance of the blank control wells}\right)}. \end{array}$$

Then, the concentrations inhibiting 50% of cell growth (IC_50_) were obtained, and these values were used for subsequent experiments. MTT assay was carried out by triplicate determination on at least three separate experiments. All data are expressed as mean ± SD.

### 2.12. Evaluation of UTR Expression by FACS Analysis and Western Blot

Untreated cells were fixed for 20 min with a 3% PFA solution and permeabilized for 10 min with 0.1% (v/v) Triton X-100 in phosphate buffered saline at room temperature. To prevent nonspecific interactions of antibodies, cells were treated for 2 h in 5% (w/v) BSA in phosphate-buffered saline and then were incubated with a specific rabbit Ab raised against UTR (1 : 50 in blocking solution, 3% BSA in TBS-Tween 0.1%) for 2 h at 37°C. After several washes, cells were incubated with a secondary goat anti-rabbit IgG-FITC (Life Technologies, Carlsbad, CA) diluted at 1 : 50 in blocking solution for 1 h at room temperature. The FITC fluorescence was measured with a FACSCalibur flow cytometer (Becton Dickinson) on 10,000 cells for each sample using the CellQuest software (Becton Dickinson). Mean fluorescence intensity (MFI) of anti-rabbit-FITC sample was considered as 100%.

Equal amounts of cell proteins from untreated cells were separated by SDS-PAGE electrophoresis (BioRad, California, USA). Proteins were electrotransferred to nitrocellulose membrane by using Trans Blot Turbo (BioRad, California, USA). Membrane were washed in TBST (10 mM Tris, pH 8.0, 150 mM NaCl, 0,05% Tween 20) and were incubated with specific MAbs and later with horseradish peroxidase-conjugated secondary antibodies. The rabbit antibodies raised against UTR and the mouse antibody raised against *α*-tubulin were purchased from Santa Cruz Biotechnology. Blots were then developed using enhanced chemiluminescence detection reagents ECL (Thermo Fisher Scientific, Rockford, IL) and exposed to X-ray film. All films were scanned by using Quantity One software (BioRad Chemi Doc).

### 2.13. Analysis of Liposome Intracellular Distribution by FACS and Confocal Microscopy

BD FACSCalibur fluorescent-activated flow cytometer and the BD CellQuest software (BD Biosciences) were used to perform flow cytometry analysis of liposome intracellular distribution. For these experiments, Bodipy-labeled liposomes were used on DU145 and PC3 cells. Bodipy is a bright, green-fluorescent dye with similar excitation and emission to fluorescein (FITC) or Alexa Fluor 488 dye. Green fluorescent-labeled liposomes were added to the medium of DU145 or PC3 cells at a fixed concentration. After 6, 24, 48, and 72 h of incubation at 37°C, removal of unattached liposomes was accomplished by washing the cells with phosphate-buffered saline at pH 7.4 for 1 min. Cells were then detached by trypsinization. The samples were subsequently washed twice, and for each sample, 10,000 cells were measured by using FACSCalibur flow cytometer (Becton Dickinson) using the CellQuest software (Becton Dickinson). After 6 h of incubation with a fixed concentration (10 *μ*M) of Bodipy-labeled liposomes, DU145 and PC3 cells were fixed for 20 min with a 3% PFA solution and permeabilized for 10 min with 0.1% (v/v) Triton X-100in phosphate-buffered saline at room temperature. To prevent nonspecific interactions of antibodies, the cells were treated for 2 h in 5% (w/v) BSA in phosphate-buffered saline and then were incubated with a specific mouse Ab raised against vimentin (1 : 50 in blocking solution, 3% BSA in TBS-Tween 0.1%) for 2 h at 37°C. After several washes, the cells were incubated with a secondary IgG goat anti-mouse-Alexa Fluor 633 (Life Technologies, Carlsbad, CA) diluted at 1 : 50 in blocking solution for 1 h at room temperature. The slides were mounted on microscope slides by Mowiol. The analyses were performed with a Zeiss LSM 510 microscope equipped with a plan-apochromat objective X 63 (NA 1.4) in oil immersion. The fluorescence of the Bodipy-labeled liposomes and vimentin was collected in the multitrack mode using BP550-625 and LP650 as emission filters, respectively.

## 3. Statistical Analysis

All data were expressed as mean values ± SD of at least three independent experiments. Statistical analysis was obtained using ANOVA, and significant differences were determined at *p* ≤ 0.01. Graphs were obtained using SigmaPlot v.11.0 (Systat Software, San Jose, CA, USA).

## 4. Results

### 4.1. Liposome Characterization

Here, we used a short peptide of eight amino acids (peptide 4-11) of the UT-II with only one binding site. In particular, a cysteine was added to the NH_2_-terminal of the peptide to provide a unique site for the conjugation with the nanocarrier. The conjugation has been carried out at the extremity of PEG chain present on the liposome surface by using maleimide groups that react with cysteine residues on ligands to form stable thioether bonds. To this aim, liposomes with DSPE-PEG_2000_-maleimide were used for the conjugation. For comparison purpose, liposomes with DSPE-PEG_2000_ were also prepared. The characteristics of LipoUT, i.e., mean diameter, PI, and zeta potential, are summarized in Table 1. The mean diameter of nonconjugated liposomes was 147.84 nm. The conjugation with the peptide significantly influenced liposome characteristics, with a slight increase of the mean diameter (168.4 nm) and a higher PI due to the presence of the peptide on the surface (Table 1). Moreover, after conjugation of UT-II, LipoUT compared with the unconjugated liposomes, showed a significant zeta potential decrease, due to the presence of peptide linked on the PEG extremity. In fact, the percentage of UT-II, present on the surface of liposomes, was about 61%. Then, the nanocarriers were loaded with a cytotoxic agent, doxo, widely used in the clinical setting. Liposomes with an internal aqueous phase containing a citrate buffer at pH 4 were prepared. The same liposomes were then purified and resuspended in a phosphate buffer at pH 7.4 to create a pH transmembrane gradient. PEGylated liposomes encapsulating doxo (Lipo-doxo) and UT-II-conjugated PEGylated liposomes encapsulating doxo (LipoUT-doxo) were prepared. The characteristics of these formulations are reported in Table 2. The encapsulation of doxo into the liposomes did not affect their characteristics. Indeed, Lipo-doxo had a mean diameter of about 150 nm with a narrow size distribution (PI ≤ 0.1). As expected, doxo encapsulation efficiency was very high in both formulations. Indeed, Lipo-doxo and LipoUT-doxo had a doxo encapsulation efficiency of 90 and 96%, respectively (Table 2). The encapsulation of doxo did not affect the *ζ* of liposomes, suggesting that negligible amount of the drug was associated with the liposome surface following purification. The different surfaces of Lipo and LipoUT, due to the presence of DSPE-PEG_2000_ and DSPE-PEG_2000_-maleimide, respectively, had only a slight influence on the doxo encapsulation process.

### 4.2. Expression of UTR on Cancer Cell Lines

In order to assess the ability of UT-II-conjugated liposomes to efficiently deliver doxo in cancer cells that express UTR, we evaluated the expression of the receptor in different human prostate (DU145, PC3, and LNCaP) and colon (WIDR and LoVo) cancer cell lines.

**Figure 1:**
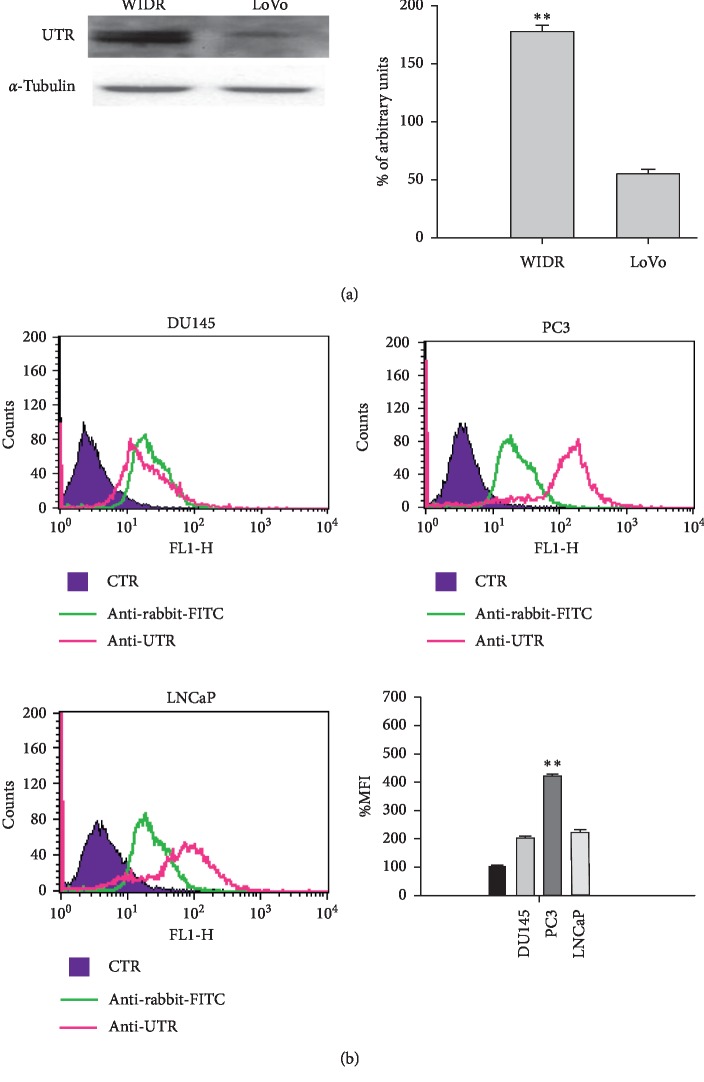
(a) Evaluation of UTR expression on colon (WIDR and LoVo) cancer cells by western blotting analysis. The expression of *α*-tubulin was assessed as loading control (left panel). The intensities of the bands were expressed as arbitrary units and detected by Quantity One software (BioRad Chemi Doc) (right panel). Error bars showed standard deviation from the mean in at least three independent experiments. Bars, SDs. ^*∗∗*^*p* ≤ 0.01 (b) Evaluation of UTR expression on DU145, PC3, and LNCaP cell lines by FACS analysis (left panel and top right panel). The % MFIs of control were calculated, as described in Materials and Methods and represented as columns (bottom right panel). The experiments were performed at least three times, and the results were always similar. Bars, SDs. ^*∗∗*^*p* ≤ 0.01.

The UTR expression was investigated by western blotting in colon cancer cells. In detail, both cell lines expressed the receptor, but the level of expression was higher in WIDR (about 4-fold) compared to LoVo (Figure 1(a)). FACS analysis was used to detect UTR expression on cell membrane of prostate cancer cells. Also, in this case, the receptor was expressed in all prostate cancer cells but, at higher levels, in PC3 (2,5-fold) and LNCaP (1,5-fold) cells compared to DU145 (Figure 1(b)).

In conclusion, UTR protein was expressed in all the assessed cell lines; the level of expression was higher in WIDR, PC3, and LNCaP cells compared with LoVo and DU145.

### 4.3. Liposome Uptake and Intracellular Distribution

To investigate if UT-II peptide conjugation to liposomes can enhance uptake and improve intracellular distribution of the formulations, FACS and confocal laser scanning microscopy (CLSM) were performed on DU145 and PC3 cells treated with fluorescent-labeled liposomes. The fluorescence associated to the cells was evaluated by FACS after 6, 24, 48, and 72 h of incubation with Bodipy-labeled liposomes, used at a fixed concentration.

**Figure 2:**
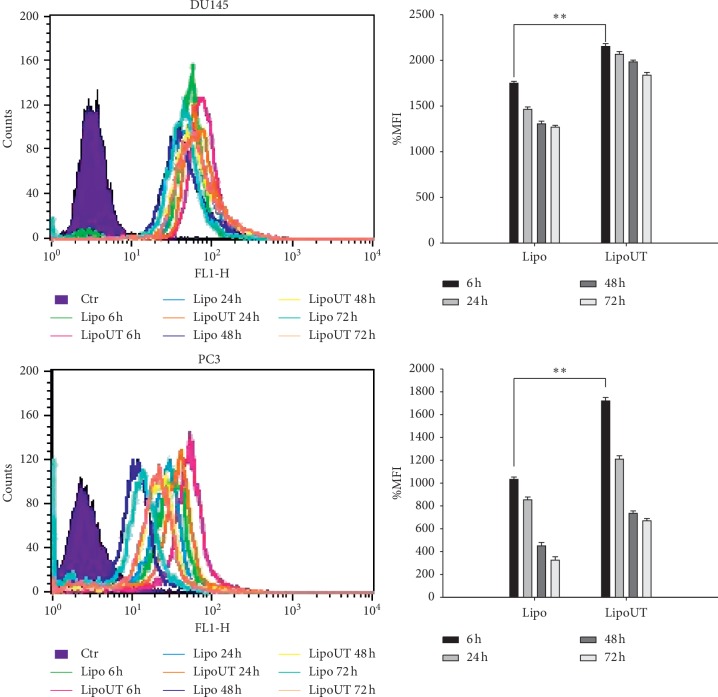
FACS analysis of DU145 and PC3 cells after 6, 24, 48, and 72 h of incubation at 37°C with Bodipy-labeled liposomes conjugated or not with UT-II (left panel). The % mean fluorescence intensity (MFI) of control was calculated, as described in “Materials and Methods” and represented as columns (right panel). The experiments were performed at least three times, and the results were always similar. Bars, SDs. ^*∗∗*^*p* ≤ 0.01.

In particular, in both cell lines (DU145 and PC3), after 6 h of incubation with LipoUT or Lipo, FACS analysis detected the % of mean fluorescence intensity (MFI) as compared with the untreated controls (Figure 2). MFI of control was considered as 100%. In both cell lines, we observed a higher internalization of LipoUT compared to Lipo but at a major extent in PC3. In detail, in DU145, that has lower UTR expression, LipoUT induced about 30% of MFI increase (2000%) compared with Lipo (1700%) while in PC3, that has higher UTR expression, LipoUT induced about 70% of MFI increase (1700%) compared with Lipo (1000%) (Figure 2). In both cases, MFIs gradually decreased in a time-dependent manner after 24 h.

**Figure 3:**
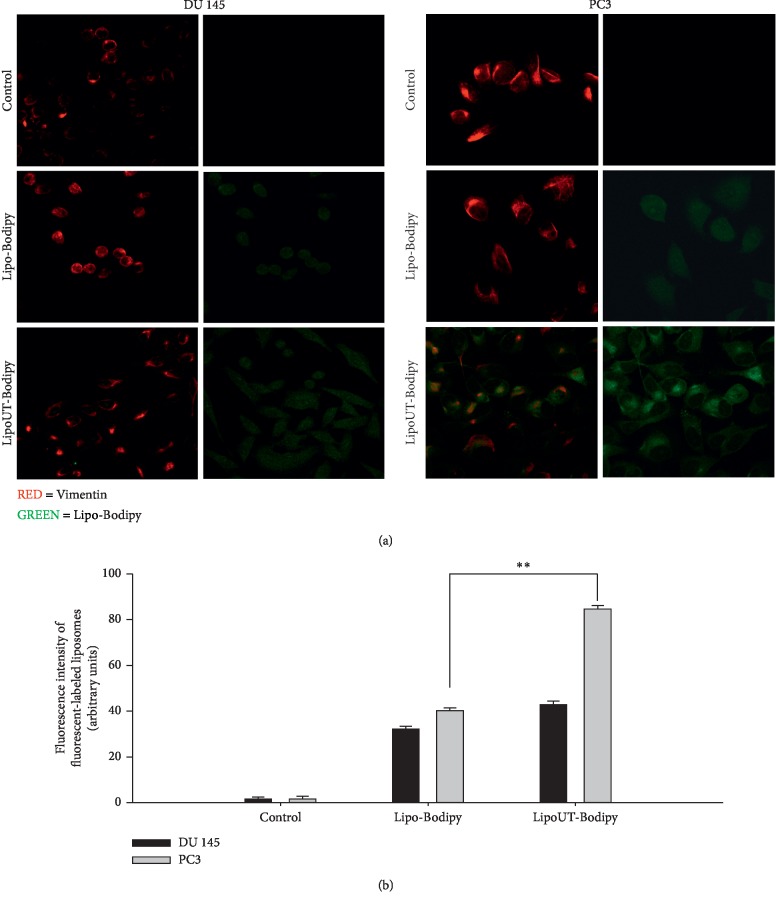
(a) Confocal microscopy images of DU145 and PC3 after 6 h of incubation at 37°C with fluorescent-labeled liposomes. Representative images of control, unconjugated liposomes (Lipo-Bodipy), and liposomes conjugated with UT-II (LipoUT-Bodipy). For each cell line, images on the left panel report that the red fluorescence associated with the Ab against vimentin; images on the right panel report the green fluorescence associated with Bodipy. The cells were visualized with a confocal microscope at magnification 100x. (b) Quantification of fluorescence intensity was reported as arbitrary units. The experiments were performed at least three times, and the results were always similar. Bars, SDs. ^*∗∗*^*p* ≤ 0.01.

The intracellular distribution of Bodipy-labeled liposomes after 6 h of incubation was studied by confocal microscopy analysis. CLSM showed a higher widespread and intense green fluorescence intensity, both in DU145 and PC3 cells incubated with UT-II-conjugated liposomes (LipoUT) compared to Lipo (Figures 3(a) and 3(b)). Moreover, when comparing the fluorescence into DU145 and PC3 cells, higher fluorescence was observed in PC3 cells (about 2-fold higher). This was likely due to the higher expression of UTR in PC3 cell line.

### 4.4. Effects of Liposome Encapsulating Doxorubicin Conjugated or Not with UT-II on Cancer Cell Lines

**Figure 4:**
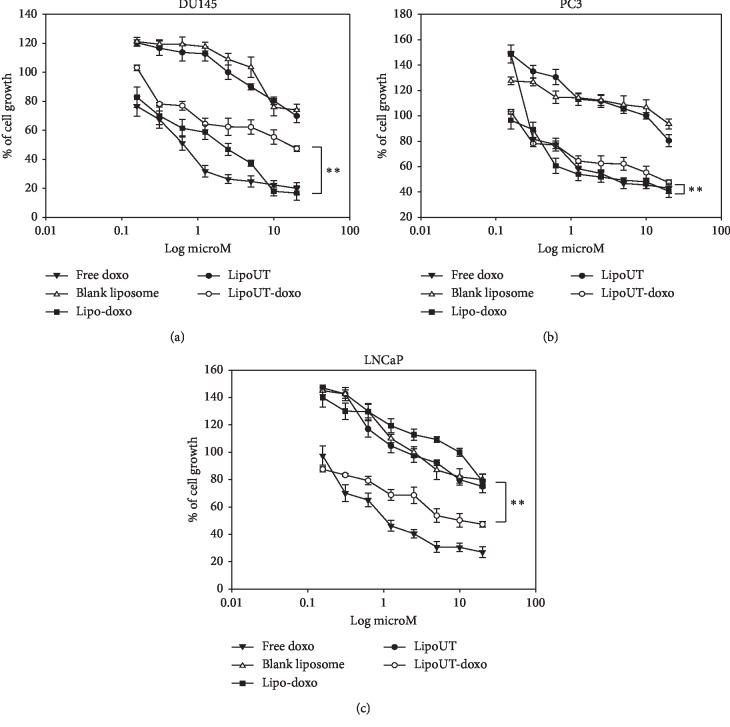
Effect of all developed formulations on prostate cancer cell lines (DU145, PC3, and LNCaP) proliferation. Prostate cancer cells were seeded in serum-containing media in 96-well plates at the density of 2 × 10^3^ cells/well. After 24 h incubation at 37°C, cells were treated with increasing concentrations of free doxo, Lipo, Lipo-doxo, LipoUT, and LipoUT-doxo (20-0,16 *μ*M) for 72 h. Cell viability was assessed by MTT assay as described in Materials and Methods. ^*∗∗*^*p* ≤ 0.01.

**Figure 5:**
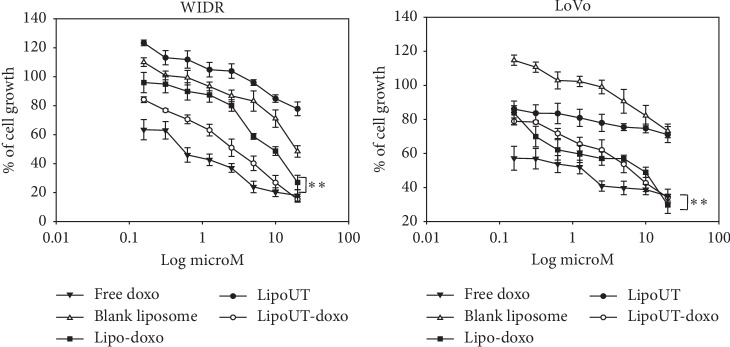
Effect of all developed formulations on colon cancer cell lines (WIDR, Lovo) proliferation. Colon cancer cells were seeded in serum-containing media in 96-well plates at the density of 2 × 10^3^ cells/well. After 24 h incubation at 37°C, cells were treated with increasing concentrations of free doxo, Lipo, Lipo-doxo, LipoUT, and LipoUT-doxo (20-0, 16 *μ*M) for 72 h. Cell viability was assessed by MTT assay, as described in Materials and Methods. ^*∗∗*^*p* ≤ 0.01.

The effects of free doxo, Lipo and liposome encapsulating doxo, and conjugated (LipoUT-doxo) or not (Lipo-doxo) with UT-II were evaluated on the proliferation of prostate (DU145, PC3, and LNCaP) and colon (WIDR and LoVo) cancer cell lines by MTT assay as reported in “Materials and Methods.” Doxo, Lipo-doxo, and LipoUT-doxo induced a dose-dependent growth inhibition in all the cell lines after 72 h, while treatment with Lipo did not produce significant cytotoxic effects (Figures 4 and 5).

**Figure 6:**
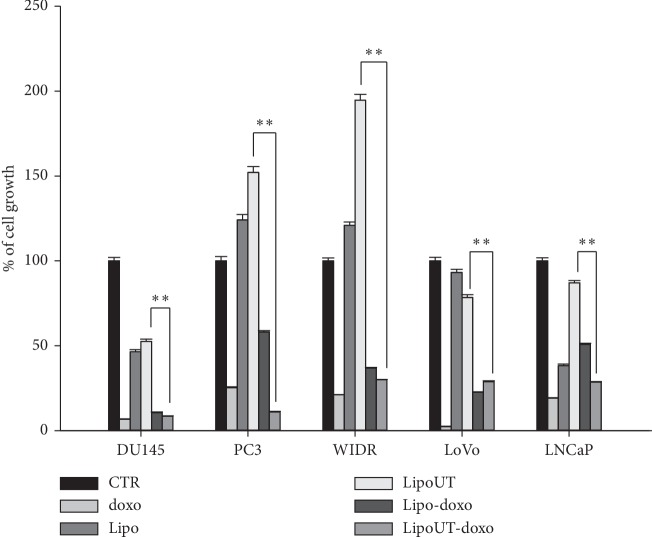
Cell proliferation of all developed formulations in prostate (DU145, PC3, and LNCaP) and colon (WIDR and LoVo) cancer cell lines. Cancer cells were seeded in serum-containing media in 96- well plates at the density of 2 × 10^3^ cells/well. After 24 h incubation at 37°C, cells were treated with 10 *μ*M of free doxo. Lipo: plain liposomes; LipoUT: PEGylated liposomes conjugated with UT-II peptide; Lipo-doxo: PEGylated liposomes-encapsulated doxo; LipoUT-doxo: PEGylated liposomes conjugated with UT-II peptide and encapsulated with doxo. Lipo, Lipo-doxo, LipoUT, and LipoUT-doxo for 72 h. Cell viability was assessed by MTT assay, as described in Materials and Methods. ^*∗∗*^*p* ≤ 0.01.

In Table 3, results are reported as concentrations inhibiting 50% of cell growth (IC_50_) after 72 h of treatment. After 72 h, IC_50_ of free doxo were equal to 0.6 *μ*M, 4 *μ*M, 1 *μ*M, 0.6 *μ*M, and 1.25 *μ*M for DU145, PC3, LNCaP, WIDR, and LoVo, respectively (see Table 3). The doxo encapsulated in liposomes (Lipo-doxo) induced a 50% growth inhibition at a concentration of 2 *μ*M, 5 *μ*M, and >20 *μ*M in DU145, PC3, and LNCaP, respectively (Table 3) and 10 *μ*M and 7 *μ*M, in WIDR and LoVo, respectively (Table 3). On the other hand, LipoUT-doxo inhibited the 50% of cell growth at a concentration of 15, 18, and 10 *μ*M, on DU145, PC3, and LNCaP, respectively, and 2.5 *μ*M and 5 *μ*M, on WIDR and LoVo, respectively. Although PC3 expresses high levels of UTR LipoUT-doxo resulted less cytotoxic than Lipo-doxo based on the evaluation of IC_50_. However, it is worthy of note that the activation of UT-II can induce cell proliferation; in fact, we found that liposomes conjugated with UT-II (LipoUT) increased cell proliferation, in particular, in PC3, LNCaP, and WIDR that overexpress UTR. On these bases, we tested all the formulations by using a very high concentration of doxo, to antagonize the effect of UT-II on cell proliferation (Figure 6). Therefore, comparing the effects of LipoUT containing doxo with empty LipoUT (15.5-fold decrease of cell growth), these effects were stronger than those induced by Lipo containing doxo compared to empty Lipo (2.16-fold decrease of cell growth) also in PC3 cells. Similarly, also on the other cell lines overexpressing UTR, the difference in cell growth inhibition was markedly increased considering the proliferative effects caused by empty LipoUT.

## 5. Discussion

Liposomal nanoparticles represent the most investigated nanotechnology platform with a plethora of novel applications still to discover. The success of liposomes as drug delivery systems is due to different factors, such as biocompatibility and biodegradability (when using phospholipids from natural source), versatility of the formulation, possibility to encapsulate drugs with different lipophilicity, design relatively easy, and possible scale-up for large production in GMP quality. Liposomes have been extensively used in cancer therapy to change drug biodistribution. In particular, liposomes allow addressing the encapsulated drug towards tissues characterized by vessels with an enhanced permeability of the endothelium, such as tumors [29, 30]. Stealth properties are obtained by modification of the liposome surface, e.g., by including polyethylene glycol (PEG). PEG has a flexible chain that occupies the space immediately adjacent to the liposome surface (“periliposomal layer”), thus excluding other macromolecules, e.g., blood plasma opsonins, from this space. Therefore, the presence of PEG prevents liposome interaction with the reticulo-endothelial system (RES) and increases the accumulation in solid tumors, due to an increased permeability of tumor vasculature [31] for the presence of fenestrated endothelium in tumor blood vessels (passive targeting) [32]. A longer circulation time results in a higher probability of liposome extravasation in tissues where capillaries are characterized by fenestrated endothelium, i.e., liver and spleen, or in tumors in which the high permeability of the vessels is associated to a reduced lymphatic drainage (enhanced permeability retention or EPR effect) [33].

Here, stealth liposomes were proposed as systems to target cancer cells overexpressing UTR and to promote drug delivery of anticancer agents, i.e., doxorubicin, into colon and prostate cancer cells. UT-II is a vasoactive peptide able to bind the UTR, a receptor involved in a number of physiological processes in different organs. This receptor has been found to be involved in proliferation, motility, and invasion of several cancer cells; among them are the colon [34] and human prostate [7] cancer cells. Moreover, UTR was overexpressed in prostate and colon cancers and its expression was correlated to the clinical outcome of neoplastic patients representing a negative prognostic factor.

The conjugation of ligands able to specifically bind receptors that are overexpressed on cancer cells to liposome surface represents an efficient active targeting strategy to enhance selectivity and efficiency of drug delivery systems. This is especially important to reduce the toxic effect of the antitumor therapies. Thus, specific ligands (i.e., transferrin, folic acid, monoclonal antibodies, and peptides) able to bind receptors overexpressed on the surface of cancer cells have been coupled on the liposome surface.

It is worthy of note that when considering proteins, such as transferrin or monoclonal antibodies, different binding sites are present on each molecules. Therefore, their conjugation with nanovectors can lead to a heterogeneous conjugate with a quality of the final product difficult to control. Based on these considerations, we used a short peptide of eight amino acids (peptide 4-11) of the UT-II with only one binding site. In particular, a cysteine was added to the NH_2_-terminal of the peptide to provide a unique site for the conjugation with the nanocarrier. The introduction of the cysteine residue was followed by purification and characterization (by reverse-phase HPLC as reported in the Materials and Methods section), thus excluding the presence of by-products, i.e., products derived by the reduction of the disulfide bridge. Moreover, the formulation proposed in this study has been developed following an optimization study to find the experimental conditions for the highest liposome/UT-II conjugation degree. In our case, a conjugation degree of about 61% was achieved (checked by HPLC). It is worthy of note that a significant percentage of the maleimide residues are not exposed on the external surface of the liposomes, thus suggesting that the percentage of conjugation obtained here is close to the highest possible. Then, the obtained nanocarriers were loaded with a cytotoxic agent, doxorubicin (doxo), widely used in the clinical setting. Doxorubicin (doxo) is an anthracycline with significant anticancer properties. Unfortunately, its use is limited by the induction of severe cardiotoxicity that occurs through poorly investigated mechanisms. Recently, it was found [35] that doxorubicin induces senescence in human vascular smooth muscle cells (VSMC) also at low calcium concentrations. On the other hand, UTR activated by UT-II produces second messengers, i.e., inositol triphosphate and diacylglycerol, which induce calcium release (Ca^2+^) and consequently activate Ca^2+^-dependent kinases (CaMKs) leading to cell proliferation [36]. In this light, UT-II/UTR complex formation in our experimental model (UT-II armed liposomes) could counteract the doxo-mediated detrimental effects on VSMC. Therefore, we selected doxo in order to demonstrate the high efficiency of liposomes in targeting UTR-expressing cancer cells but, on the basis of the histotype of the selected tumors, other drugs with lower cardiovascular side effects could be encapsulated. The first liposomal formulation encapsulating doxo, Doxil, was approved by FDA in 1995. The encapsulation of doxorubicin in stealth liposomes showed a significant decrease of the cardiotoxicity and enhanced the efficacy of doxorubicin, due to a higher accumulation in tumors, in drug-resistant tumors, compared with free doxorubicin [12].

Doxo was also chosen because it can be easily encapsulated into liposomes by the well-known *remote-loading* method [27]. This technique allows the achievement of high drug encapsulation efficiency by using a transmembrane pH gradient. Moreover, doxo is a fluorescent molecule easily assessable in cells. We preferred the use of indirect method for a faster determination of doxorubicin and conjugated peptide, due to the presence of the new lipid-peptide conjugate in the formulation. Indeed, the newly synthesized PEGylated lipid conjugated with UT-II was considered a source of bias due to the not well-known solubility in organic solvents (cosolubilization of lipid mix with doxorubicin) and to a difficult-to-predict interaction with surfactants such as Triton X-100 (e.g., method described by A. Fritze et al.) [37]. Thus, a rapid determination of the doxorubicin encapsulation was achieved by an indirect method, as previously reported by others [38]. Similarly, the dithiotreitol cleavage should be validated for the newly-synthesized PEGylated lipid conjugated with UT-II, while we used an indirect determination by the HPLC method largely validated in previous studies on the same peptide [10]. The encapsulation of doxo into the liposomes did not affect their characteristics. As expected, doxo encapsulation efficiency was very high in both formulations. The encapsulation of doxo did not affect the *ζ* of liposomes, suggesting that negligible amount of the drug was associated to the liposome surface following purification. On the other hand, the conjugation of UT-II to liposomes did not significantly affect the mean diameter of the liposomes when compared to Lipo-doxo but produced a PI increase and a significant zeta potential decrease. Despite this, liposomes with a mean diameter of about 169 nm can still be considered suitable for intravenous administration, especially when considering the PI of about 0.2. Moreover, after conjugation of UT-II with liposomes, LipoUT compared with the unconjugated liposomes showed different *ζ*, due to the presence of peptide linked on the PEG extremity. In fact, the percentage of UT-II, present on the surface of liposomes, was about 61%.

In order to assess the ability of UT-II-conjugated liposomes to efficiently deliver doxo in cancer cells that express UTR, we evaluated the expression of the receptor in different human prostate (DU145, PC3, and LNCaP) and colon (WIDR and LoVo) cancer cell lines.

UTR protein was expressed in all the assessed cell lines; the level of expression was higher in WIDR, PC3, and LNCaP cells compared with LoVo and DU145. To investigate if the UT-II peptide conjugation to liposomes can enhance uptake and improve intracellular distribution of the formulations, FACS and confocal laser scanning microscopy (CLSM) were performed on DU145 and PC3 cells treated with fluorescent labeled liposomes. In particular, we found that cell uptake of Bodipy-labeled liposomes in PC3 and DU145 was higher for LipoUT than the not armed counterparts but at major extent in PC3 cells that express higher UTR levels.

Many efforts have been made to increase selectivity and the efficacy of the liposome delivery. The strategy of active targeting has been successfully used to specifically deliver drugs to cancer cells. Moreover, it is possible to target receptors able to be internalized upon binding with the ligands, thus promoting the intracellular delivery of the drug associated with the ligand, with consequent increase of the cytotoxicity [14, 15]. UTR is expressed in several human tissues, such as heart, brain, aorta, and kidney. It has been reported [39] that UT-II dissociation from UTR is an irreversible mechanism. In fact, when an excess of unlabeled UT-II was added in human rhabdomyosarcoma cells, only 15% of [125I] UT-II was dissociated after 90 min. Internalization experiments performed with radioactive-labeled UT-II (125I-UT-II) showed that about 70% of activated UTR receptors on the cell membrane of human embryonic kidney 293 cells were internalized through endocytosis mechanisms within 30 minutes (half life [th]: 5.6 ± 0.2 minutes). The internalization of UT-II/UTR complex was dynamin-dependent and arrestin-independent. After UT-II dissociated from UTR, the receptor quantitatively recycled back to the plasma membrane within 60 minutes (th 31.9 ± 2.6 minutes) [40]. The low rate of dissociation and the continuous externalization of UTR determine the long-lasting UT-II-mediated effects. UTR, for its specific internalization characteristics, is a suitable molecule for targeting purposes because it is easily internalized, rapidly dissociates from the ligand, and is also recycled on the cell membrane avoiding downregulation effects due to the binding with its own ligand.

On these bases, the effects of free doxo, Lipo, LipoUT-doxo, and Lipo-doxo were evaluated on the proliferation of prostate (DU145, PC3, and LNCaP) and colon (WIDR and LoVo) cancer cell lines. LipoUT-doxo was more active than Lipo-doxo on the growth inhibition of cells that overexpressed UTR (LNCaP and WIDR), while in LoVo and DU145 cell lines, the activity was similar to or lower than that one of Lipo-doxo, respectively. Although PC3 express high levels of UTR, LipoUT-doxo resulted to be less cytotoxic than Lipo-doxo based on the evaluation of IC_50_. These findings seem in contrast with observation of confocal microscopy in which higher liposome uptake was observed in UTR overexpressing cells. However, it is worthy of note that the activation of UTR by the agonist UT-II can induce cell proliferation as described in our previous work [4]; in fact, we found that empty liposomes conjugated with UT-II (LipoUT) increased cell proliferation, in particular, in PC3, LNCaP, and WIDR that overexpress UTR. On these bases, we tested all the formulations by using a very high concentration of doxo, to antagonize the effect of UT-II on cell proliferation. Based upon these considerations, the growth inhibitory effects obtained with LipoUT-doxo was underestimated due to the counteracting proliferative effect caused by UT-II expressed on the surface of the liposomes. Therefore, comparing the growth of LipoUT containing doxo with empty LipoUT, the effects of the former were much stronger than those induced by Lipo containing doxo as compared to empty Lipo in PC3 cells. Similarly, also on the other cell lines overexpressing UTR, the difference in cell growth inhibition was markedly increased considering the proliferative effects caused by empty LipoUT. In these conditions, the effects of LipoUT containing doxo were more potent also than those induced by free doxo in the cells overexpressing UTR.

Different anticancer drugs have been incorporated in nanocarriers to address several issues, such as the poor solubility of drugs, the drug instability in biological fluids, and serious side effects due to the drug accumulation into the healthy tissues together with low concentration at the action site [41]. More recently, nanomedicine has been proposed also to overcome the multidrug resistance (MDR) of cancer cells to chemotherapeutic agents that promote an overexpression or suppression of certain molecular pathways with consequent treatment failure [42–44]. Similarly, liposomes and, in general, lipid nanovectors, modified with specific ligands, were used to enhance drug delivery into tumors. In particular, the access of drugs to the central nervous system (CNS) is hampered by the blood-brain barrier (BBB) formed by brain endothelial cells connected by tight junctions. However, in presence of brain tumors, the increase of the intracranial pressure leads to partial alternation of the BBB permeability. Thus, colloidal particles can access, in these conditions, the CNS. On the other hand, modification of nanocarrier surface with specific endogenous or exogenous ligands, e.g., transferrin, promoted enhanced BBB crossing, also in case of unaltered endothelium [45, 46]. On these bases and according to the obtained data, targeting tumor-related receptors could represent a successful drug delivery strategy against tumors. Additional in vivo studies are needed to better investigate the efficiency and specificity of these new delivery systems, and we would have benefit of transparent in vivo models, such as zebrafish embryos, to evaluate fluorescent-labeled liposome biodistribution [47–51].

## 6. Conclusion

In conclusion, we have developed liposomes conjugated with UT-II (LipoUT) for efficient targeting of cancer cells that overexpress UTR and promoting drug delivery of an anticancer agent, i.e., doxorubicin, into colon and prostate cancer cells. We demonstrated that LipoUT-doxo was more active than Lipo-doxo on the growth inhibition of cells that overexpressed UTR (PC3, LNCaP, and WIDR). Moreover, we found that cell uptake of Bodipy-labeled liposomes in PC3 and DU145 was higher for LipoUT. Taken together, the data obtained in this work suggest that the encapsulation of doxo in UT-II-targeted liposomes potentiated its delivery in UTR overexpressing cells and could represent a new tool for the targeting of prostate and colon cancer.

## Figures and Tables

**Table 1:** Mean diameter, PI, and zeta potential (*ζ*) of plain liposomes (Lipo) and liposomes conjugated with UT-II (LipoUT).

Formulation	Diameter (nm ± SD)	PI ± SD	*ζ* (mV ± SD)	UT-II on the surface of liposomes (%)
Lipo	147.8 ± 6.90	0.098 ± 0.03	−19.58 ± 5.90	—
LipoUT	168.4 + 9.55	0.203 + 0.07	−26.10 + 4.34	61%

**Table 2:** Mean diameter, PI, and zeta potential (*ζ*) of liposomes encapsulating doxo conjugated (LipoUT-doxo) and unconjugated (Lipo-doxo).

Formulation	Encapsulation efficiency doxo (%)	Diameter (nm ± SD)	PI ± SD	*ζ* (mV ± SD)	UT-II on the surface of liposomes (%)
Lipo-doxo	90	148.9 ± 8.27	0.102 ± 0.06	−18.76 ± 3.74	—
LipoUT-doxo	96	190.9 ± 17.42	0.247 ± 0.11	−28.89 ± 2.15	60%

**Table 3:** IC_50_ values after 72 h treatment with free doxo, doxo encapsulated in liposomes (Lipo-doxo), and doxo encapsulated in liposomes conjugated with UT-II (LipoUT-doxo).

Cell lines	Doxo (IC_50_*μ*m)	Lipo-doxo (IC_50_*μ*m)	LipoUT-doxo (IC_50_*μ*m)	UTR expression
DU145	0.6 ± 0.016	2 ± 0.013	15 ± 0.076	Low
PC3	4 ± 0.031	5 ± 0.018	18 ± 0.089	High
LNCaP	1 ± 0.011	>20 ± 0.091	10 ± 0.045	High
WIDR	0.6 ± 0.018	10 ± 0.087	2.5 ± 0.018	High
LoVo	1.25 ± 0.013	7 ± 0.064	5 ± 0.013	Low

## Data Availability

The data used to support the findings of this study are available from the corresponding author upon request.
